# Online comprehension across different semantic categories in preschool children with autism spectrum disorder

**DOI:** 10.1371/journal.pone.0211802

**Published:** 2019-02-11

**Authors:** Rita Barone, Concetto Spampinato, Carmelo Pino, Filippo Palermo, Anna Scuderi, Anna Zavattieri, Mariangela Gulisano, Daniela Giordano, Renata Rizzo

**Affiliations:** 1 Child Neurology and Psychiatry Unit—Department of Clinical and Experimental Medicine; University of Catania, Catania, Italy; 2 CNR-Institute for Polymers, Composites and Biomaterials IPCB, Catania, Italy; 3 Department of Electrical, Electronic and Computer Engineering; University of Catania, Catania, Italy; 4 Biostatistics—Department of Clinical and Experimental Medicine; University of Catania, Catania, Italy; 5 School of Medicine, University of Catania, Catania, Italy; University of Amsterdam, NETHERLANDS

## Abstract

**Background:**

Word comprehension across semantic categories is a key area of language development. Using online automated eye-tracking technology to reduce response demands during a word comprehension test may be advantageous in children with autism spectrum disorder (ASD).

**Objectives:**

To measure online accuracy of word recognition across eleven semantic categories in preschool children with ASD and in typically developing (TD) children matched for gender and developmental age.

**Methods:**

Using eye-tracker methodology we measured the relative number of fixations on a target image as compared to a foil of the same category shown simultaneously on screen. This online accuracy measure was considered a measure of word understanding. We tested the relationship between online accuracy and offline word recognition and the effects of clinical variables on online accuracy. Twenty-four children with ASD and 21 TD control children underwent the eye-tracking task.

**Results:**

On average, children with ASD were significantly less accurate at fixating on the target image than the TD children. After multiple comparison correction, no significant differences were found across the eleven semantic categories of the experiment between preschool children with ASD and younger TD children matched for developmental age. The ASD group showed higher intragroup variability consistent with greater variation in vocabulary growth rates. Direct effects of non-verbal cognitive levels, vocabulary levels and gesture productions on online word recognition in both groups support a dimensional view of language abilities in ASD.

**Conclusions:**

Online measures of word comprehension across different semantic categories show higher interindividual variability in children with ASD and may be useful for objectively monitor gains on targeted language interventions.

## Introduction

Autism Spectrum Disorder (ASD) is a common neurodevelopmental disorder characterized by severe social communication deficits and stereotyped, repetitive behaviors. ASD is often accompanied by delayed development of verbal communication. Language ability is highly variable—from completely absent to almost preserved language skills—and it impacts treatment approaches and long-term prognosis [1]. Language development at an early age has been extensively studied in preschool children with ASD compared to typically developing (TD) toddlers and same-aged preschoolers with developmental delay. Most findings concur on shared elements of language development in children with or without ASD. In common with typical development is a considerable variability in language acquisition, although some studies support the existence of differences such as higher proportion of severe language delay and greater variation in vocabulary growth rates in ASD [2–5].

Language development relates to several factors including cognition and adaptive functioning, severity of ASD symptoms, and non-verbal communication skills such as gestures. Receptive and expressive language and gestures increase with increasing non-verbal mental age in children with ASD as a group but are reduced in comparison to TD children with the same non-verbal mental age suggesting that cognitive level does not explain all language variability in children with ASD [2, 6–7].

The most used tools for language assessment in children with ASD include direct measures and caregiver reports. A prospective study using the Mullen Scales of Early Learning [8] as a direct method to evaluate language comprehension showed lower scores in children with ASD as compared to TD peers by 14 months of age. In addition, the ASD group obtained lower scores than the group of children with language delay at the age of 24 months [9]. It is worth mentioning that standardized direct assessment of receptive language in children with ASD may be challenging and influenced by several factors including absence of a pointing response and general difficulties understanding the pragmatics of test situations. Moreover, direct assessment measure of language may be affected by testing demands including attention, following instruction and producing a clear response [10]. These factors are particularly relevant when examining children with severe impairments, as seen in minimally verbal children with ASD.

Indirect assessment by parent report of receptive vocabulary on the MacArthur-Bates Communicative Development Inventory (MCDI) [11] showed that MCDI scores in children with ASD at ages 2 and 3 years were predictive of outcome at age 9 years thus supporting the use of the MCDI parent-report as a quick and informative tool to measure early verbal skills in children with ASD [12].

More recently, online measures of eye movements have been increasingly used in young children with ASD acquiring English to provide a direct assessment of verbal comprehension [13]. Online measures of receptive language based on automated eye-tracking technology reduce the demand on the child's response by simply asking to look at the image that is being named in a *looking-while-listening* (LWL) task. In this way, online measures in those children with ASD having difficulties in executing commands or with defective pointing use can facilitate measuring receptive language ability [14–15]. Both in young TD children [16] and in late-talking toddlers [17] real-time verbal processing relates to vocabulary level.

A few language studies have applied automated eye tracking technology to measure receptive language in children with ASD [18–22]. In older preschoolers with ASD at age 5, words acquired earlier in life were processed more quickly than words acquired later, pointing to similarities in the pattern of language development between children with ASD and TD children [19]. Recently, an eye-tracking test of word comprehension was applied in a comprehensive study comparing multiple methods including technology-based assessment methods of receptive language in minimally verbal children and adolescent with ASD. Results showed high interindividual heterogeneity in receptive language and across assessment methods. Consequently, in minimally verbal children with ASD the use of individualized approaches that may include methods based on eye-tracking or touch-screen responding is envisaged [22].

Comprehension of words across semantic categories is a key area of language development. Lexical composition was investigated in TD children acquiring different languages and late-talkers [23–26] while it was somewhat less explored in young children with ASD [2,5,27]. Pivotal studies on vocabulary composition in TD children were conducted using parent report checklists such as the MCDI [23] and the Language Development Survey (LDS) [24–25]. Overall, the findings show that both TD and late-talkers children vary in their rate of lexical acquisition and there are similarities in vocabulary composition across various languages, including Italian [26].

Two studies on lexical composition on parent-report checklists reported that in children with ASD acquiring English semantic category distributions were similar to those of TD children [2] and to those of late talkers [27] with comparable vocabulary sizes. However, in either studies, no details about the specific words acquired were reported. Importantly, one investigation on lexical composition at the word level found that children with ASD acquired essentially the same words as younger TD children suggesting that their lexical development is more delayed than deviant [5].

The present study aims to extend current knowledge on lexical comprehension in children with ASD exploring the possibility to employ less demanding tools in an experimental task based on looking-behavior. For this purpose, we focused on understanding lexical composition using a direct and validated measure of word comprehension by an eye-tracking task probing “online” comprehension.

The first aim of the current study was to evaluate comprehension of words across various semantic categories, in preschool children with ASD acquiring Italian compared to TD children. We combined an indirect measure based on the MCDI with an online procedure of word recognition designed on the same categories examined on the MCDI. In particular, we considered 102 words belonging to the eleven semantic categories listed in the MCDI to develop an eye-tracking procedure based on the LWL paradigm [28]. We measured the relative number of fixations on a target image as compared to a foil of the same category shown simultaneously on screen and we considered it as a measure of word understanding. Then, we evaluated for each semantic category the association between the MCDI completed by the parents and direct assessment of word understanding by the online procedure. The second aim was to consider the influence of age, cognitive levels, gesture use and ASD severity in early word comprehension using different semantic categories when measured by online methods. As a whole, we used an online measure of word comprehension across semantic categories in children with ASD and TD children learning a language different than English. This study using an online procedure aims to determine whether there are differences in early lexical development between ASD and TD children. We also aim to determine if findings from studies which use English are applicable to other languages such as Italian.

## Methods

### Participants

All procedures performed in studies involving human participants were in accordance with the ethical standards of the institutional and/or national research committee and with the 1964 Helsinki declaration and its later amendments or comparable ethical standards. Informed consent was obtained from all individual participants included in the study.

Twenty-four children with a diagnosis of ASD (mean age: 43.5 months; range 24–61; 5 girls) were evaluated. Diagnosis of ASD was obtained according to strict criteria and standardized diagnostic tests using Autism Diagnostic Interview-Revised (ADI-R) [29] and Autism Diagnostic Observation Schedule (ADOS) [30]. Among ASD individuals, exclusion criteria were the presence of an associated monogenic disease (i.e., Tuberous Sclerosis, Fragile-X syndrome), or the occurrence of proven neurological and/or sensorial defects (i.e., cerebral palsy, epilepsy, impaired vision, hearing impairment). As control group, twenty-one TD children (mean age: 31.4 months; range 21–42; 3 girls) were recruited in a local kindergarten in Catania. It has to be noted that in several studies on the semantic composition of the vocabulary based on offline measures, preschoolers with ASD and TD children were matched on vocabulary-size. We considered that online word comprehension could be influenced by different cognitive functions besides language itself. Moreover, nonverbal cognition has long been considered a concurrent predictor of receptive and expressive language development [2,31]. Based on these arguments, in the present study ASD and TD participants were matched on developmental age (DA). The Griffith scale equivalent age (months) of performance subscale (non-verbal cognition) was considered as a measure of DA [32]. The mean DA was 34.2 and 33.4 for the TD group and the ASD group, respectively. After assuming equal population variances by Levene’s test, a statistical significance T-Test for equality of means showed that the two groups did not differ in DA (t(43) = -.19, p = .851, d = .062). The TD group was significantly younger than the ASD group (t (43) = 3.08, p = .004, d = 1.01) (Table 1). All participants were from Italian speaking families. Written informed consent was obtained from the parents of all participants. The current study was part of an overall larger study aimed at identifying markers, predictors and developmental trajectories of ASD. The larger overall study was approved by the local ethics committee at University Hospital Policlinico Catania.

**Table 1:** Participant characteristics and language measures.

	ASD (n.24)		TD (n.21)		
	M (SD)	Range	M (SD)	Range	p
**Chronological age (months)**	43.5 (10.2)	24–61	31.4 (8.41)	21–42	.*004*
**Developmental age**°	33.4 (13.1)	18–49	34.2 (13.2)	21–44	.851
Total word comprehension*	166 (95.8)	45–380	206 (90.9)	114–379	.241
Total action-gestures*	33.6 (14.1)	10–58	45.2 (10.2)	30–63	.059
**Checklist word measure§**	68.7 (24.4)	23–94	76 (18.2)	47–102	.437

M: mean. SD: standard deviation.

°The Griffith scale equivalent age (months) of performance subscale (non-verbal cognition) was considered as a measure of developmental age.

*Raw number of words comprehended and action-gestures from the CDI.

§Caregivers from 17 and 19 participants of the ASD and TD groups respectively completed the vocabulary checklist.

Italics represents statistically significant differences (Bonferroni corrected p value).

### Standardized measures

#### Cognitive development and ASD symptoms assessment

The Griffith Mental Development Scale (GMDS) is used to assess the child development from birth to 8 years. The six areas of development measured by the scale include: Locomotor, Personal-Social, Hearing and Language, Eye and Hand Coordination, Performance and Practical reasoning. GMDS has been widely validated for developmental assessment in young children with ASD [32]. The Calibrated Severity Score (CSS) from 4 to 10 was used as a measure of Autism severity and it was calculated based on ADOS raw scores and chronological age as described [33]. The calibrated scores indicate a classification of non-spectrum (1–3), ASD (4–5) and autism (6–10). In children with TD the lifetime version of the Social Communication Questionnaire (SCQ) [34] was used to rule out autistic behaviors.

#### Language measures

The MCDI Word and Gestures Forms are parent-completed report forms that allow clinicians to collect information on child’s understanding of early vocabulary items belonging to specific semantic categories in children aged 8–18 months. The Italian version of MCDI was used as a measure of lexical ability in all participants [35]. The questionnaire includes: a checklist of 408 words divided into 19 semantic categories including items for nominal (e.g., animals, vehicles, toys), routines (e.g., people, games), predicates (i.e., verbs, adjectives) and function words (e.g., pronouns, prepositions, quantifiers). For the analysis of semantic repertoire, we considered noun and action categories. For the noun category, all the answers given by the parents to the list of 284 items referring to the 10 different semantic groups *(animals*, *vehicles*, *toys*, *food*, *clothing articles*, *body parts*, *furniture items*, *household objects*, *outside things and people)* were calculated. Likewise, for the action category, all the answers given by the parents to the list of 55 verbs were considered. We measured the parent report of vocabulary comprehension by raw number of words understood, as the children in the study were older than normative groups [36]. In addition, raw number of the MCDI Gestures and Actions checklist, including communicative and/or symbolic action-gestures (maximum score: 63), was considered. Each parent was asked if the child habitually produced the actions and gestures included in the questionnaire. Standardized test to directly assess the receptive lexical knowledge as *Peabody Picture Vocabulary Test*–Revised (*PPVT*-R) [37] could not be administered to most of the children with ASD because of difficulties with testing procedures. Therefore results from *PPVT-R* were not included in the data analysis.

#### Caregiver vocabulary checklist

In order to assess the actual child understanding of the words used in the LWL task, we developed a checklist of all the 102 words used in the experimental task. The list consisted of nouns and action words, selected from the MCDI, Words and Gesture form. We asked the caregivers to check off which words were understood by the child. The list of words included a 1-point rating scale (Yes, No) to provide an assessment about the child’s comprehension of the listed words. Caregivers from 17 and 19 participants of the ASD and TD groups respectively completed the vocabulary checklist.

### Spoken word recognition task

In the present study an eye tracker was used with the aim of analyzing single spoken word recognition in children with ASD diagnosis. To this aim, two images of the same semantic category appeared simultaneously, side by side, on the screen at the beginning of each trial. Each word was used as target and foil within the same category. A total of 102 different target words (the same word used for both conditions) were presented in 102 different trials distributed over 11 categories to all participants. According to the MCDI the following semantic categories were considered: *animals*, *vehicles*, *toys*, *food and drink*, *clothing*, *body parts*, *furniture and rooms*, *household objects*, *outside things*, *people and actions*. The content of the 11 categories was established in terms of frequency of use of the different words according to the Italian MCDI. Moreover, the number of words used was proportional to the words’ number listed in the MCDI for each category. Based on both criteria we used for each category a number of words ≥ 7 and ≤ 13, representative of the words with frequency ≥ 20% reported on the Italian MCDI. The list of words used and their frequency is reported in S1 Appendix. Of note, we used a lower number of words (no. 5) for “toys” because this category includes only 8 words in the MCDI. As concerns the action category, we used 12 words representative of those with the highest frequency among 55 action words listed on the MCDI.

### Visual and auditory stimuli

The visual stimuli were non- copyrighted digital color photos collected from the web showing the target words. All selected pictures were selected by consensus of all researchers involved in the study. All images were considered prototypical images with only one single object or figure and assigned to one word. Image pairs were presented against a blank background. Children sat on their parent’s lap approximately 60 cm in front of the eye tracker monitor (17''), with eye-level corresponding to the center of the screen, in a sound-attenuated room. Image pairs were automatically displayed every 1000 ms for a duration of 5000 ms on a black screen. Areas of interest (AOI) were defined by the edges containing the photographs. The expected/average visual angle measured 15° vertically and 11.5° horizontally at the distance of 60 cm. In order to avoid possible variation in the performance due to position of each category block along the task, the order of category presentation was randomly assigned for each participant while the order of image presentation (trials) within each category was maintained the same across participants. We choose not to randomize all trials into blocks of mixed categories because we aimed to measure word understanding in different categories. To account for possible image salience, within each category, each image served as target and then as foil. Target and foil images were counterbalanced for side presentation (left or right) across trials within each category. The audio stimuli matched on the target image had been recorded by a female native Italian speaker and it was automatically played at 80 dB volume. The target word was pronounced with the images as: “Look at the *Ball*”, followed by the word *“Ball”* only. Since it is not grammatically correct to say “Look at the (verb)”, for action category the audio stimuli was the bare verb, repeated two times. The duration of the audio stimuli ranged from 500 to 650 ms accounting for the variable length of words in the task.

### Eye-tracking task

Specific eye-tracking software was developed for the visual word tasks. The software tool allows customization of specific tests by setting the number of visual word categories, visualization times and AOI definition. Consistent with the aforementioned task, the tool displays two images (target and distractor) for a time period. Eye-tracking data was acquired with a Tobii T60 binocular eye tracker (Tobii Technology AB, Danderyd, Sweden). This noninvasive eye-tracking system uses infrared sensors to track both eyes, to a rated accuracy of 0.5°, sampled at 60 Hz. It was calibrated for each participant using a 5-point calibration. Gaze location for each time sample was categorized as target, distractor, or neither. The target audio word was played 1 s after the visual stimulus onset. We measured both number and time of fixation and used as accuracy measure the number of fixations on the target image after the word onset in each trial, divided by total fixation numbers to both target and foil in the time window. We obtained the same accuracy levels when considering the proportion of looking time to the target image divided by total looking time to both target and foil. The time window was computed as the looking time from 300 to 1800 ms after the onset of the second instance of the target word. This time window was chosen because previous research showed that 200 ms is the time needed to plan the eye movement after an auditory stimulus [22]. Gaze shifts occurring 1800 ms after word onset were excluded because they are less clearly in response to the target word [22]. The participants’ looking behavior was monitored by the investigator on a separate computer in real time. By this procedure, the investigator could stop the experiment in case the child’s gaze was not displayed on the screen or it was over fixed on one side of the screen. In our study, 102 trials were divided in eleven blocks corresponding to the number of study categories. Thus, although the trials were always 5 s in duration, an interval time (≤ 3 m) was interposed between category blocks to help the participant reorient attention to the screen to minimize eye-movement data loss. In the case of failing to obtain usable gaze data in a category block, no more than one additional attempt was made for that category. In the ASD group, nine children necessitated one additional attempt to obtain usable gaze data in the following categories: *animals* (no. 3), *vehicles* (no.3), *clothes* (no.1), *body parts* (no.1), *outside objects* (no.1) *and people* (no. 1). Among TD participants, one child required a second attempt in four categories (*vehicles*, *toys*, *clothes and outside things*) while seven children required it in one or two categories: *animals* (no.3), *vehicles* (no.2), *body parts* (no. 1) and *people* (no.1).

### Data analysis

Independent t-tests were computed to compare offline measures of word comprehension between the two groups. To identify variables associated with offline and online word comprehension univariate and bivariate analyses were performed whilst controlling for chronological age, non-verbal DA and autism severity (CSS). Two-tailed p < .05 were considered statistically significant. To control for the inflation of the Type I error rate due to multiple comparisons, the Bonferroni correction was applied. Due to lack of normal distribution, non-parametric analyses were used to analyze between-group differences (ASD vs TD; Mann-Whitney U) and within-group differences of online accuracy (Kruskal-Wallis test). All data were analyzed with the SPSS 20.0 software for Windows (SPSS Inc., Chicago, IL, USA.) As a measure of lexical comprehension, we calculated the proportion of fixations to the target picture (accuracy) in each trial after the onset of the target word divided by total number of fixations to both target and foil in the time window. Following the widely accepted approach to interpreting fixation patterns in a looking-while-listening (LWL) procedure [28,38], we considered the participants comprehended the word when fixated more times and longer at the target image than the foil.

We included eye-gaze data where the child did look at the target or foil for at least 0.5 sec, corresponding to 10% the duration of the trial (5 s). Therefore, based on the total looking time, we could not include all trials for all participants. In the ASD group, 6 of 24 participants (16%) contributed with a variable amount of data (66%-94% of all trials) while in the TD group, 4 of 21 participants (19%) contributed to 93% to 95% of the trials. The percentage of usable gaze data did not differ significantly between category blocks (all p >0.5).

## Results

### Standardized measures of cognitive development and autism severity

Mean, SD and range of performance DA, as a measure of non-verbal mental development, are reported in Table 1. Among TD children, eight subjects (38%) had global developmental quotient (DQ) (developmental age/chronological age×100) in the low-normal range (median 73; range: 70–76). We assume that the presence of children with low-normal DQ explains the lack of a significant correlation between DA and chronological age in the control group (r (n = 21) = .223,^,^ p = .331).

Fourteen children (58%) within the ASD group exhibited cognitive delays relative to age expectations with variable DQ ranging from 46 to 73 (median: 62). Ten children with ASD (41%) had normal global development (median DQ: 82; range: 79–91). In participants with ASD, cognitive level demonstrated wide variability without correlation to participants’ age (r (*n* = 24) = .128, p = .631). Six ASD participants were classified with total CSS of 4–5 consistent with Autism Spectrum Disorder classification. Eighteen participants had a CSS between 6 and 10 consistent with an Autism classification. Autism severity score was not related to participants’ age (r (n = 24) = —.385, p = .273). Chronological age was not significantly different between ASD children with and without cognitive delay (z = 1.210, p = .235) whereas severity of autism symptoms was significantly higher in children with lower cognitive levels (z = 2.766, p = .0005). Likewise, the vocabulary-size measured by word checklist was significantly lower in children with ASD and cognitive delay (z = 4.045, p = .00001).

### Lexical comprehension and production of action and gesture from the MCDI; Caregiver report checklist

Table 1 shows the descriptive analyses (means, standard deviations and ranges) of parent reported receptive vocabulary (number of words understood) and actions and gestures identified on the MCDI for all participants. Most of the children (75%) with ASD had a language impairment of variable degree related to word comprehension and production compared to reference values for chronological age [36]. The mean receptive vocabulary size was 166 words with high interindividual variability (45 to 380). TD children were significantly younger than ASD children and had a lexical ability as expected for their chronological age [36]. T-tests showed no group differences in parent reported receptive vocabulary (t(41) = -1.19, p = .241, d = .98), gesture use (t(41) = -1.97, p = .059, d = .90) and vocabulary checklist (t(34) = -0.789, p = .437, d = .80) between the ASD and TD groups (Table 1).

Pearson correlation analyses were conducted between measures of word comprehension and clinical variables (Table 2). Total word comprehension including nouns and predicates was significantly related to non-verbal DA (r (n = 24) = .770, p = .002). We measured the action and gesture produced from the MCDI as a measure of non-verbal communication in children with ASD. Out of a total of 63 gestures considered, the mean total number of communicative and/or symbolic action-gestures reported by parents on the MCDI was 33.6, indicating on average, a reduced gesture production with high inter-individual variability (10 to 58) in children with ASD. Total gesture produced was significantly related to word comprehension (r (n = 24) = .788, p = .001) as well as to DA (*r* (n = 2) = .754, p = .003). Moreover, a significant, negative association between gesture production and CSS total was observed (r (n = 24) = —.720, p *=* .005). Word comprehension was not significantly associated to autism severity measured by CSS (r (n = 24) = -.498, p = .080) (Table 2).

**Table 2:** Results of Pearson correlation analyses in participants with ASD (correlation values in children with TD in parenthesis).

	DA	Word comprehension	Gesture produced	Online Accuracy	Autism Severity
**Chronological Age**	.128 (.223)^a^	.421 (.390)	.385 (.419)	.283 (.312)	-.385
**Developmental Age**		.770** (.672*)	.754** (.496)	.602*(.670*)	-.665**
**Word Comprehension**			.788** (.605*)	.718** (.610*)	-.498
**Gesture Produced**				.704** (.470)*	-.720**
**Online Accuracy**					-.672*

^a^ correlation values in participants with TD in parenthesis.

Developmental age (DA) was measured on performance subscale at GMDS. Word comprehension and gesture produced were measured by MCDI raw scores. Autism severity was measured by calibrated ADOS severity scores.

*p< .05

**p< .01(uncorrected significance levels).

### Eye-tracking task

On average, children with ASD were significantly less accurate at fixating on the target (median 0.55; range 0.48–0.61) than the TD group (median 0.59; range 0.53–0.65) (z = -3.938, p = .0001). We compared the accuracy levels of ASD and TD groups for each semantic category. No statistically significant differences were found across the eleven semantic categories of the experiment between preschool children with ASD and chronologically younger TD children matched for DA (significance level: .05/11; Bonferroni corrected significant p-value: .004). (**Table 3**).

**Table 3:** Online accuracy of ASD and TD control groups for each semantic category. The number of participants contributing to each category is reported.

Category (no.words)	ASD participants			TD participants			Mann-Whitney U Test	
	Mean ± SE	Median (range)	(no.)	Mean ± SE	Median (range)	(no.)	Z	p-value
***Vehicles (7)***	0.55 ± 0.02	0.56(0.26–0.79)	(24)	0.59 ± 0.01	0.60 (0.27–0.98)	(19)	-0.697	0.486
***Animals (12)***	0.57 ± 0.01	0.55(0.34–0.86)	(24)	0.57 ± 0.01	0.56(0.45–0.69)	(21)	-0.611	0.541
***Body Parts (8)***	0.54 ± 0.01	0.54(0.32–0.73)	(24)	0.54 ± 0.02	0.55(0.34–0.65)	(20)	-0.147	0.883
***Clothes (7)***	0.52 ± 0.02	0.53(0.15–0.79)	(23)	0.58 ± 0.01	0.58(0.40–0.72)	(21)	-2.201	0.028
***Food and Drinks (13)***	0.56 ± 0.02	0.57(0.23–0.80)	(24)	0.59 ± 0.01	0.59(0.35–0.70)	(21)	-0.978	0.328
***Furniture and Rooms (8)***	0.53 ± 0.02	0.54(0.28–0.74)	(22)	0.61 ± 0.04	0.65(0.16–0.98)	(19)	-2.161	0.031
***Household Objects (11)***	0.60 ± 0.02	0.60(0.39–0.84)	(23)	0.62 ± 0.03	0.64(0.35–0.97)	(21)	-0.657	0.511
***Outside Things (9)***	0.60 ± 0.02	0.59(0.43–0.85)	(23)	0.64 ± 0.02	0.64(0.47–0.96)	(21)	-1.011	0.312
***People (8)***	0.57 ± 0.02	0.57(0.32–0.92)	(23)	0.61 ± 0.02	0.58(0.33–0.92)	(21)	-1.087	0.277
***Toys (5)***	0.48 ± 0.09	0.47(0.20–0.70)	(23)	0.53 ± 0.03	0.52(0.23–0.78)	(21)	-1.162	0.245
***Actions (12)***	0.50 ± 0.04	0.51(0.15–0.79)	(23)	0.59 ± 0.07	0.58 (0.35–0.89)	(21)	-2.668	0.021

We found that various categories with the highest accuracy values were the same in the two groups including *household objects*, *outside things*, *people*, *animals*, *food and drinks and vehicles*. Meanwhile, the category *toys* displayed the lowest accuracy levels in both groups although we consider that such a result might reflect an insufficiency of the number of words used for this category.

It is also important to note that three semantic categories including *furniture and rooms*, *clothes and actions* were among those with the highest mean online accuracy in TD children but not in those with ASD.

**Fig 1:**
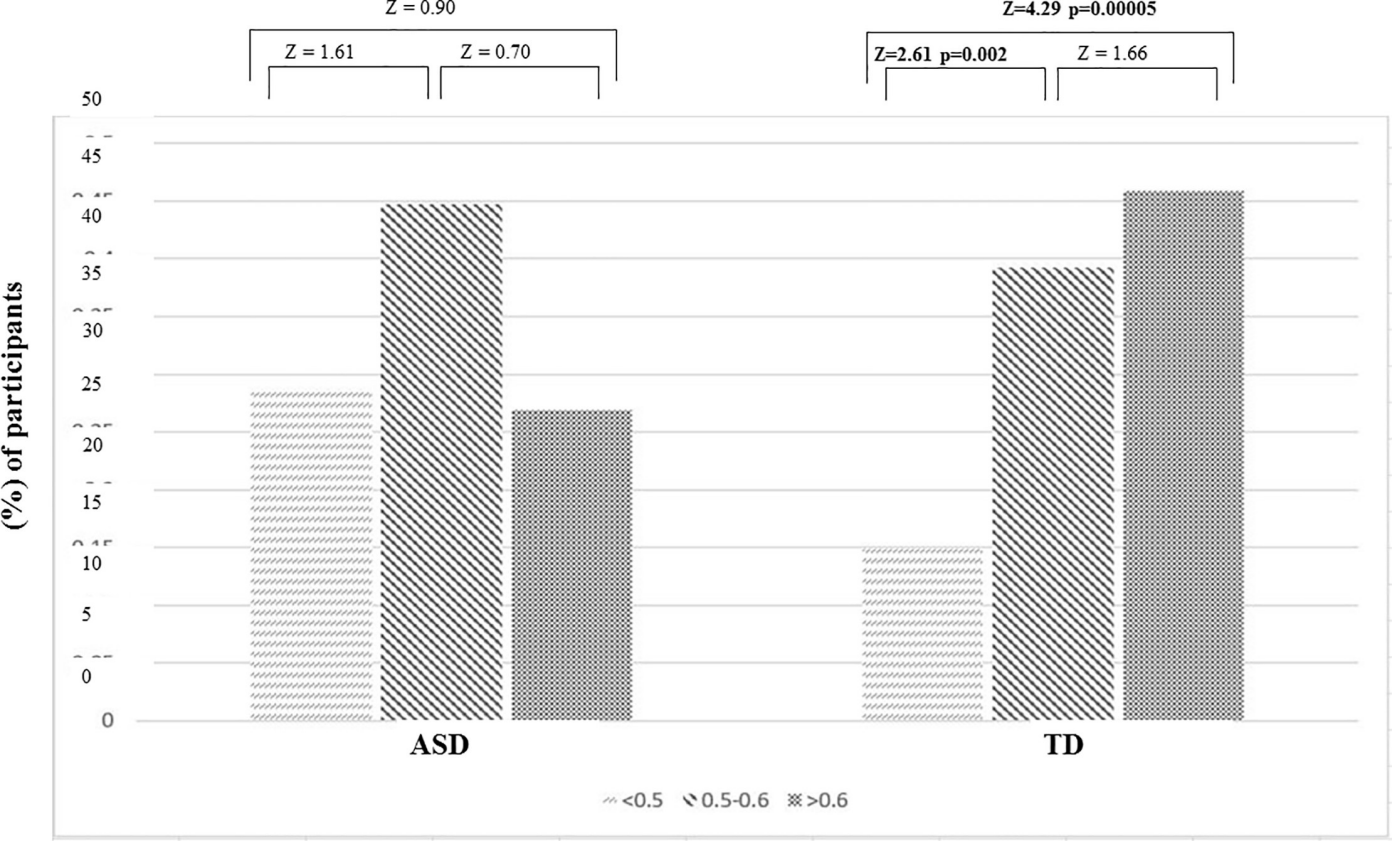
Comparison of the proportion of participants within groups in three online accuracy ranges: 1) looked at target <0.5; 2) looked at target between 0.5 and 0.6, and 3) looked at target ≥ 0.6. The z-values of the Kruskal-Wallis test between pairs are given on top of the charts and statistically significant values are highlighted in bold.

Online accuracy was very variable across participants (0.15 to 0.98) suggesting that one child might spend equal time fixating on the target and the foil whereas another child might spend clearly more fixations at the target (S2 Appendix). Therefore, to better understand possible heterogeneity among individual children we computed intra-group differences in the accuracy levels. For computational purposes, we considered a 3-level stratification of the online accuracy obtained for all participants: (1) looked at target <0.5; (2) looked at target between 0.5 and 0.6, and (3) looked at target ≥ 0.6. We compared the proportion of participants within group for each accuracy level (Fig 1). In the TD group, the proportion of participants with accuracy <0.5 was significantly lower than those with accuracy ≥0.6 (z = 4.29 p = .00005) or accuracy between 0.5 and 0.6 (z = 2.61 p = .002). The results show that most children with TD homogeneously performed at higher accuracy levels (Fig 1). On the other hand, no significant differences among the proportion of children with ASD in the three accuracy levels were found, suggesting a higher intra-group variability of online accuracy in the ASD group.

Finally, we computed accuracy levels for each category in ASD group with cognitive delays and in those with normal global development to examine whether particular patterns of responding in the eye-tracking task could be identified. When performing comparison for individual category, we found a significant difference for the *outside* category (z = 2.108, p = .035) and the *action* category (z = 2.454, p = .007).

### Correlation among offline language measures and clinical variables with online accuracy

In the TD group there were significant positive correlations among online accuracy and number of words understood (r (n = 21) = .610, p = .021) and number of produced gestures (r (n = 21) = .470, p = .034) respectively. The findings show that children with larger vocabulary looked proportionally more at the target than their peers with smaller vocabularies (Table 2). Likewise, in the ASD group there was a significant correlation among offline and online measures of word comprehension with all the correlations being moderate to large thus providing an index of validity for the experimental task (Table 2). We found that, in general, the two measures (online accuracy and comprehended words on CDI) converged in all the semantic categories. When they did not converge, it was most probable that the parent’s overestimated comprehension, with the exception of two very severely affected children (minimally verbal) whose comprehension might have been underestimated in four and two categories, respectively.

Gesture use and non-verbal cognition were positively associated with online and off-line measures of verbal comprehension, supporting an association among preverbal communication skills (gestures), offline comprehension and developmental age with online word comprehension measures in children with ASD (Table 2). Partial correlations showed that age had very little influence in accounting for the relationship between accuracy and cognitive level, since it remained significant whilst controlling for age (r (22) = .608, n = 24, p = .012). Whilst controlling for autism severity, there was no evidence of a relationship between accuracy and cognitive level (r (22) = -.345, n = 24, p = .190), suggesting that ASD symptoms might hinder online accuracy on this task despite cognitive levels. At the same time, when controlling for cognitive level, the correlation between individual accuracy and autism severity was weakened (r (22) = -.501 n = 24, p = .048). The findings suggest that individual cognitive level influenced the relationship between online accuracy and autism severity (CSS) in children with ASD.

## Discussion

In the present study we analyzed key areas of early language acquisition for all children, namely the comprehension of different categories of words. Previous studies using offline measures showed that children with ASD did not differ from TD children [2] and late-talkers [27] in the semantic category distributions of their lexicons suggesting a delayed but not deviant pattern of word learning. A pivotal study by Rescorla and Safyer (2013) examined the composition of lexicon at word level in sixty-seven children with ASD aged 1;6–5;11 in comparison to TD children by using the LDS. It was found that ASD and TD children had high percentage use scores for the same words, independent of vocabulary size supporting the notion of language delay rather than deviance in language comprehension [5]. In the current study we investigated whether online accuracy in word comprehension differed as a function of the semantic category of the target words in preschoolers with ASD compared to TD children matched on DA. We considered 102 words (nouns and actions) belonging to eleven semantic categories listed in the MCDI. In each category we included words varying in frequency of use in order to examine lexicon distribution across categories. Actually, we could not rule out *a priori* that the children with ASD were acquiring some atypical or idiosyncratic words in their lexicon. On average, children with ASD were significantly less accurate at fixating on the target than the TD children across the eleven categories of the experiment. However, no statistically significant differences were found for each semantic category between groups, after multiple comparison correction. The lack of group differences related to semantic category between the ASD and TD children suggests a delay rather than deviance in terms of the lexical composition of the children’s receptive vocabularies.

In order to clarify whether semantic category-related patterns of word recognition emerged we highlighted those categories with the highest or lowest online accuracy in either or in both groups. In the present study, various categories with the highest accuracy values were the same in the two groups including *household objects*, *outside things*, *people*, *animals*, *food and drinks and vehicles*. Such results by online comprehension of Italian words across semantic categories are consistent with earlier research based on offline comprehension of words conducted in children acquiring English. In fact, previous studies comparing lexical composition in TD children and late talkers showed they tended to learn the same words [39]. Moreover, both TD children and late talkers acquired words with high percentage use scores even in studies examining languages other than English, thus supporting cross-linguistic similarities in lexical composition development [26–27]. On the other hand, lexical development in young children with ASD has been less investigated. In one study comparing lexical development at word-level no significant group differences were found for semantic category distribution in children with ASD and TD children with small vocabulary size (1–49 words). In addition, it was found that ASD and TD children had high percentage use scores for the same words, independently from the vocabulary size (< 50 or ≥50) [5].

Although we did not compute comprehension at single word level, we would emphasize that the majority of words used in the present study were among those with highest-frequency of use in children acquiring Italian (S1 Appendix). Noteworthy, most of these words were reported as highest-frequency words in English-learning TD children and in those with ASD [5] as well as in TD children acquiring Italian [26]. These words represent a variety of semantic categories such as *foods*, *body parts*, *vehicles*, *outdoors*, *clothes*, *animals*, *household and actions*. The current study in Italian children suggests that characteristics of word acquisition between TD and ASD children are similar in the Italian and English languages, thus supporting cross-linguistic similarities in language acquisition mechanisms found in children with ASD. However, it is important to note that in the present study three semantic categories including *furniture and rooms*, *clothes and actions* were among those with highest mean online accuracy in TD children but not in those with ASD. These differences could be attributed to linguistic and non-linguistic features of ASD that will be briefly outlined. In line with differences in language development, we found a higher intra-group variability of online word recognition across semantic categories in children with ASD compared to TD, that is consistent with greater variation in vocabulary growth rates in ASD [2–3]. The asymmetry in online measures of words belonging to different semantic categories might reflect typical differences in language processing that appear related to words’ age of acquisition in the TD population, thus supporting current arguments for a dimensional view of language abilities in children with ASD. Differences in picture naming and conceptualization in Italian speaking TD children is linked to words’ age of acquisition that, in turn, depends on familiarity, typicality, and word frequency [40]. Moreover, several studies indicated a prominent role of age-of-acquisition in many language processing tasks—for example, word recognition, picture naming, and lexical decision tasks [41–43]. Age of acquisition and word speed processing on an online task were computed in children with ASD, showing that words typically learned earlier in life were processed more quickly [19]. As to non-linguistic feature of ASD eventually explaining differences with TD children, it has to be noted that any inferences about online comprehension should take into account the heterogeneity in basic attentional processes in children with ASD. Attention may be differently driven by different classes of words or visual referents [44]. Also, some children with ASD may have atypical visual attention mechanisms, difficulties in disengaging attention as well as intersensory processing abnormalities which could influence performance on the online language measure [45].

The second aim of the current study was to consider the influence of clinical variables in early noun comprehension in children with ASD as measured by an online method using different semantic categories. For this purpose, we evaluated the effects of cognitive, non-verbal communication (gestures) and behavioral correlates on the lexical measurements, using indirect and online word recognition tasks. We found that online accuracy in ASD and TD groups is significantly associated with the concurrent number of words understood reported on the MCDI. The results suggest that the eye-tracking procedure provides evidence of word comprehension in the studied children. In recent times, online measures of receptive language have received progressively more attention for evaluating receptive vocabulary and language processing speed in children with ASD. Venker et al. [19] tested children with ASD between the ages of 3 and 6 using online-measures of language comprehension and processing; they found that online accuracy was related to vocabulary comprehension on the MCDI three years earlier. Thus eye-gaze measurements of lexical knowledge appear to reflect the words understood as measured by the MCDI concurrently (present study) and reflect the vocabulary-size on the MCDI retrospectively [19]. One prominent difference between the eye-tracking procedure in the current study and those used in other studies that focus on single-word comprehension is that the current study used a higher number of words (102 divided in 11 categories). Previous eye-tracking analyses using the LWL method included 6 to 84 nouns (e.g., Venker et al. [19] tested 8 words; Bavin et al. [20] included 18 words, and Plesa Skwerer et al. [22] analyzed 84 words distributed in 3 blocks). Given that the performance on this task depends on attention monitoring we analyzed and reported the proportion of usable data trials contributed by each participant (S2 Appendix). We could obtain a sufficiently reliable data set from each participant by adopting an experimenter-controlled administration, as previously described in a pivotal study using online language in minimally verbal children with ASD [22].

In the present study we found a direct relation between gestures and online accuracy on word recognition. Gestures act as a framework for early language development and predictor of progress in verbal language, supporting the relationship between motor programs associated to actions and gestures and language [46]. Importantly, in children with ASD at early stages of language development, receptive language was predicted by concurrent gesture use as well as non-verbal cognitive ability and joint attention [31]. The findings support the existence of common factors, in addition to the vocabulary level, related to online and offline language comprehension in preschool children with ASD and TD. Children with severe autism, as determined from their ADOS-CSS, were less probable to look at the target even after controlling for their cognitive functioning, most likely because both of these factors control children’s more general attentional abilities, which could relate directly to their performance on the LWL task. Likewise, Bavin et al. (2014) focused on the extent to which autism severity affected word understanding in an online procedure and showed a negative association between language processing and autistic behavior. In our group of children with ASD between 24 and 61 months, age did not relate significantly with any variables tested, including language, cognitive raw scores and eye-gaze accuracy of word recognition. Such findings might reflect the severe extent of language impairment of some participants, since the group included subjects representing the most severe end of the spectrum (minimally verbal children) [13].

Some limitations of the current study should be noted. First, although this study provides description on online word comprehension across semantic categories, no information was acquired at word level. Actually, we could evaluate lexicon distribution across semantic categories in individual participants but we have not obtained information on single word comprehension. Further studies are required to define how word frequency in different semantic categories might impact online language measures of word comprehension. One more limitation of the present study was that we purely explored online accuracy across multiple semantic categories. Therefore, the lack of significant differences between groups could possibly be attributed to lack of power as multiple comparison correction was applied. Further studies may consider narrowing the analyses on a limited number of specific theoretically interesting comparisons to reduce the chance of Type I error.

The current study suggests that TD children perform significantly better than children with ASD (p < .05) in some semantic categories (*clothes*, *furniture and rooms and actions*). Moreover, evaluation of accuracy levels in ASD children, showed that those with normal global development (DQ) performed significantly better in *outside* category and also in *action* word recognition. Thus, when considering online accuracy of action recognition, TD children performed better than children with ASD as a group. In turn, children with ASD and normal DQ were more accurate in recognizing words that describe actions than children with ASD and developmental delay. In this regard, a cross-linguistic analysis of Italian versus English early lexical development (18–23 months) showed that late-talkers and vocabulary-size matched younger children have higher percentages of nouns compared to verbs among the words with highest reported use in each language. Moreover, in both languages, noun dominance decreases as vocabulary size increases [26].

To the best of our knowledge, the present study first used online measure of receptive language across semantic categories in children with ASD learning a language different than English. As a whole, our study advances existing knowledge about lexical development in children with ASD and suggests some similar patterns in early lexical development across different kinds of learners (i.e. TD and ASD) and across languages. In summary we have shown: 1) a greater inter-individual variability of online word recognition across different semantic categories among preschool children with ASD, with respect to chronologically younger TD matched for DA, consistent with greater variation in vocabulary growth rates in ASD. General issues related to delay, regression or compensation of developmental trajectory may result in high inter-individual variability at different ages observed in ASD 2) in both groups we found direct effects of cognitive levels, vocabulary levels and gesture productions on online word recognition, supporting current knowledge about a dimensional view of language abilities in ASD. The current study shows the use of an online method to objectively measure word comprehension across semantic categories in preschool children with ASD including those with more severe language disturbance and autism symptoms. Online measures may become functional to accurately monitor gains on targeted language interventions. However, we emphasize that performance on these tasks is highly dependent on attention monitoring and should be analyzed in relation to the proportion of usable data trials contributed by each participant, as recommended [22]. In conclusion, our investigation of online comprehension across semantic categories in preschoolers with ASD acquiring Italian suggests that language skills varied widely but were generally delayed. The findings concur with results of studies based on offline evaluations disentangling lexicon composition in children with ASD acquiring English and support cross-linguistic similarities in lexical acquisition [5]. Further studies are required to understand how the frequency of words in different semantic categories might influence online measures of word comprehension in children with ASD.

## Supporting information

S1 Appendix List of words used in the online procedure organized by MCDI semantic category. Percentage use score according to the Italian MCDI is reported.
(XLSX)

S2 Appendix Individual performances on eye-tracking task. (XLSX)
